# Sample size calculation for clinical trials: the impact of clinician beliefs

**DOI:** 10.1054/bjoc.1999.0902

**Published:** 1999-12-08

**Authors:** P M Fayers, A Cuschieri, J Fielding, J Craven, B Uscinska, L S Freedman

**Affiliations:** Cancer Division, MRC Clinical Trials Unit, 222 Euston Road, London, NW1 2DA, UK; University Department of Surgery, Ninewells Hospital and Medical School, Dundee, DD1 9SY, UK; Department of Surgery, Queen Elizabeth Hospital, Birmingham, B15 2TH, UK; Kingstown General Hospital, St Vincents, West Indies; Department of Mathematics, Statistics and Computer Science, Bar Ilan University, Ramat Gan, 52900, Israel

**Keywords:** sample size, prior beliefs, clinicians', opinions, clinical trial

## Abstract

The UK Medical Research Council (MRC) randomized trial of gastric surgery, ST01, compared conventional (D1) with radical (D2) surgery. Sample size estimation was based upon the consensus opinion of the surgical members of the design team, which suggested that a change in 5-year survival from 20% (D1) to 34% (D2) could be realistic and medically important. On the basis of these survival rates, the sample size for the trial was 400 patients. However, this trial was exceptional in the way that a survey of surgeons' opinions was made at the start of the trial, in 1986, and again before results were analysed but after termination of the trial in 1994. At the initial survey, the three surgeons from the trial steering committee and 23 other surgeons experienced in treating gastric carcinoma were given detailed questionnaires. They were asked about the expected survival rate in the D1 group, anticipated difference in survival from D2 surgery, and what difference would be medically important and influence future treatment of patients. The consensus opinion of those surveyed was that there might be a survival improvement of 9.4%. In 1994, prior to closure of the trial, and before any survival information was disclosed, the survey was repeated with 21 of the original 26 surgeons. At this second survey, the opinion of the trial steering committee was that 9.5% difference was more realistic. This was in accord with the opinion of the larger group, which remained little changed since 1986. The baseline 5-year D1 survival was thought likely to be about 32%, which corresponded closely to the actual survival of recruited patients. Revised sample size calculations suggested that, on the basis of these more recent opinions, between 800 and 1200 patients would have been required. Both surveys assessed the level of treatment benefit that was deemed to be sufficient for causing surgeons to change their practice. This showed that the 13% difference in survival used as the study target was clinically relevant, but also indicated that many clinicians would remain unwilling to change their practice if the difference is only 9.5%. The experience of this carefully designed trial illustrates the problems of designing long-term, randomized trials. It raises interesting questions about the common practice of basing sample size estimates upon the beliefs of a trial design committee that may include a number of enthusiasts for the trial treatment. If their opinion of anticipated effect sizes drives the design of the trial, rather than the opinion of a larger community of experts that includes sceptics as well as enthusiasts, there is likely to be a serious miscalculation of sample size requirements. © 2000 Cancer Research Campaign


					In 1986 the UK Medical Research Council (MRC) designed a
randomized clinical trial (ST01) comparing D1 versus D2 surgery
for operable gastric cancer. At that time D1 surgery was the
conventional surgical procedure for gastric surgery in the West,
while D2 radical surgery with extended lymph node dissection
was standard practice in Japan. Japanese reports, based upon non-
randomized studies, indicated probable major survival benefits
associated with D2 surgery. Five-year survival of (Japanese) D2
patients was nearly double that of (Western) D1 patients
(Maruyama et al, 1987). However, it was unknown whether these
results were partly or entirely due to factors such as patient selec-
tion and rigorous staging classification. The MRC trial was
designed to address these questions, using randomization to ensure
an unbiased comparison. The principal end point of the study was
5-year survival. One noteworthy aspect of this trial is that it repre-
sents one of the earliest examples of a purely surgical randomized
trial in cancer, and to date there are still very few trials comparing
two or more alternative methods of surgery.
METHODS
Design of ST01 trial
The ST01 trial design team, which later formed the trial steering
committee, included three consultant surgeons with an interest in
treatment of gastric cancer. Patients with resectable advanced
gastric cancer were to be randomized between the standard UK
operation (variations on the D1 theme) and the more radical D2
resection practised by members of the Japanese Research Society
for Gastric Cancer. Eligible patients were defined as potentially
curative S0-2
P0
H0
N0-2
, that is stage I-III gastric cancer, without
positive infracolic aortic nodes. All patients underwent staging
laparotomy to confirm potentially curative disease. Eligible
consenting patients fit for either D1 or D2 surgery were then
randomized centrally (over the phone) within the same operating
session. More extensive details of the staging and surgical proce-
dures have been published elsewhere (Cuschieri et al, 1996, 1999).
When the trial was being designed in 1985-86, there was
considerable uncertainty about the range of plausible and
Sample size calculation for clinical trials: the impact of
clinician beliefs
PM Fayers1
, A Cuschieri2
, J Fielding3
, J Craven4
, B Uscinska1
and LS Freedman5
1
Cancer Division, MRC Clinical Trials Unit, 222 Euston Road, London NW1 2DA, UK; 2
University Department of Surgery, Ninewells Hospital and Medical
School, Dundee DD1 9SY, UK; 3
Department of Surgery, Queen Elizabeth Hospital, Birmingham B15 2TH, UK; 4
Kingstown General Hospital, St Vincents,
West Indies; 5
Department of Mathematics, Statistics and Computer Science, Bar Ilan University, Ramat Gan 52900, Israel
Summary The UK Medical Research Council (MRC) randomized trial of gastric surgery, ST01, compared conventional (D1) with radical (D2)
surgery. Sample size estimation was based upon the consensus opinion of the surgical members of the design team, which suggested that a
change in 5-year survival from 20% (D1) to 34% (D2) could be realistic and medically important. On the basis of these survival rates, the
sample size for the trial was 400 patients. However, this trial was exceptional in the way that a survey of surgeons' opinions was made at the
start of the trial, in 1986, and again before results were analysed but after termination of the trial in 1994. At the initial survey, the three
surgeons from the trial steering committee and 23 other surgeons experienced in treating gastric carcinoma were given detailed
questionnaires. They were asked about the expected survival rate in the D1 group, anticipated difference in survival from D2 surgery, and
what difference would be medically important and influence future treatment of patients. The consensus opinion of those surveyed was that
there might be a survival improvement of 9.4%. In 1994, prior to closure of the trial, and before any survival information was disclosed, the
survey was repeated with 21 of the original 26 surgeons. At this second survey, the opinion of the trial steering committee was that 9.5%
difference was more realistic. This was in accord with the opinion of the larger group, which remained little changed since 1986. The baseline
5-year D1 survival was thought likely to be about 32%, which corresponded closely to the actual survival of recruited patients. Revised sample
size calculations suggested that, on the basis of these more recent opinions, between 800 and 1200 patients would have been required. Both
surveys assessed the level of treatment benefit that was deemed to be sufficient for causing surgeons to change their practice. This showed
that the 13% difference in survival used as the study target was clinically relevant, but also indicated that many clinicians would remain
unwilling to change their practice if the difference is only 9.5%. The experience of this carefully designed trial illustrates the problems of
designing long-term, randomized trials. It raises interesting questions about the common practice of basing sample size estimates upon the
beliefs of a trial design committee that may include a number of enthusiasts for the trial treatment. If their opinion of anticipated effect sizes
drives the design of the trial, rather than the opinion of a larger community of experts that includes sceptics as well as enthusiasts, there is
likely to be a serious miscalculation of sample size requirements. (c) 2000 Cancer Research Campaign
Keywords: sample size; prior beliefs; clinicians' opinions; clinical trials
213
British Journal of Cancer (2000) 82(1), 213-219
(c) 2000 Cancer Research Campaign
Article no. bjoc.1999.0902
Received 28 October 1998
Revised 22 February 1999
Accepted 8 July 1999
Correspondence to: PM Fayers. E-mail: PFayers@hotmail.com
clinically important survival benefits that might follow D2
surgery. However, this information was necessary for the estima-
tion of sample size (Fayers and Machin, 1995). From past
experience in UK, the baseline survival at 5 years for such patients
undergoing a D1 resection was judged to be 20%. This estimate
was obtained as a consensus opinion of the three surgical members
of the ST01 design team. Japanese figures, based upon observa-
tional (non-randomized) studies suggested a large survival
advantage to D2 surgery, but this could have been influenced by
other factors. These included (a) earlier diagnosis, through
screening and public awareness; (b) stage migration, since the
extensive surgery enabled more rigorous classification and detec-
tion of poor-prognosis patients; and (c) physical fitness or other
characteristics of the patients, since Japanese patients tend to be
less obese and younger than Western patients. Thus members of
the ST01 design team had varying opinions as to the magnitude of
any survival advantage in favour of D2. The ST01 team was also
aware that D2 surgery, being far more extensive than D1, was
likely to be accompanied by increased post-operative morbidity
and mortality. Thus there would have to be reasonably large
long-term survival advantages for D2 to become the treatment of
choice. For sample-size calculations we therefore assumed the
5-year survival would be 20%, and that the target treatment
difference would be about 13%.
The protocol specified that the main analysis would be based
upon a comparison of survival using the log-rank test. With a
baseline survival rate of 20%, 400 patients (200 per treatment arm)
would enable an improvement of 13.5% (to 33.5%) to be detected
with a 5% P-value and 90% power (Machin et al, 1997).
Therefore, in 1986 the ST01 trial was launched with a target
sample size of 400 randomized patients. Since the eventual
analysis would depend upon comparison of survival rates, it would
only be possible to analyse the data after sufficient `events', in this
case deaths, had occurred (Fayers and Machin, 1995). The
protocol specified that detailed analysis would be deferred until
after the accumulation of 250 deaths in the trial.
First survey of surgeons' opinions
The sample size estimation depends crucially upon the magnitude
of the anticipated treatment effect. Whilst 400 patients suffice to
be reasonably confident (90% power) of detecting a 13% improve-
ment, one would need nearly 700 patients for a 10% improvement.
On the other hand, only about 200 patients are needed for detec-
tion of a 20% improvement. The baseline survival rate (20%
5-year survival in D1 patients) is less critical to the sample size
estimation, but it is particularly important to be confident about the
magnitude of the treatment effect that one hopes to detect. The
expectations regarding the benefit of D2 surgery were investigated
through a survey of surgeons.
Prior to launching the trial, eight surgeons experienced in
gastric surgery, including the three surgical members of the
steering committee, were individually interviewed, and another 18
intending trial participants completed a postal questionnaire. The
214 PM Fayers et al
British Journal of Cancer (2000) 82(1), 213-219 (c) 2000 Cancer Research Campaign
Figure 1 Extract from the survey questionnaire
survey asked `what differences in 5-year survival rate would influ-
ence you to use D1 or D2?' This emphasizes one often overlooked
aspect of clinical trials, namely that the role of a clinical trial
should not be merely to establish treatment differences, but should
be to influence medical practice. The surgeons knew that D2
surgery was more extensive, carried extra risk of complications,
and demanded extra resources. If D2 surgery offers no survival
benefit, D1 should be the operation of choice. If D2 offers substan-
tial survival benefit, it should be the treatment of choice in suitable
patients. But there may be a range of small survival benefit within
which surgeons remain uncertain as to whether D1 or D2 is appro-
priate. This is called the `range of equivalence'. Therefore,
surgeons could specify a range of values within which they would
remain uncertain whether to use D1 or D2, and if the results of the
trial suggested that the survival difference between D1 and D2 lay
within this range the surgeon would have no strong preference for
either form of surgery. This approach probably reflects clinical
thinking more closely than if one demanded a single value for the
treatment difference, above which treatment 1 is preferred and
below which treatment 2 is favoured. A clear decision can only be
made if the survival difference is found to lie outside the range of
equivalence. If the results are more extreme in favour of D2
surgery, the surgeon would prefer D2. Similarly, if results are
outside the range of equivalence in the opposite direction, they
would choose D1.
A clinical trial should also be realistic. It would be of little rele-
vance to design and conduct a clinical trial on the basis of seeking
larger survival benefits than can reasonably be expected to be
present. Therefore, the surgeons were asked to indicate what
difference in survival they expected would emerge if many
patients were given the two treatments. They had to indicate a
range of values, and weight their beliefs. For example, a surgeon
could have indicated an expected 5-year survival advantage to D2
surgery of 20% or more. The surgeon could then indicate with
decreasing confidence that it could be above 25%, or even 30% or
more. In this manner a `prior distribution' of beliefs for each
surgeon was constructed.
The survey also included various other questions concerning the
expected baseline 5-year survival for D1, their level of experience
with D2 surgery, and factors influencing their answers (such as the
perceived risk of post-operative complications associated with
D2). Figure 1 shows extracts from the questionnaire used in the
survey.
Second survey of surgeons' opinions
Before the trial was completed, and before any results had been
revealed, a second survey was carried out. The same surgeons,
where traceable, were approached. They were asked to complete
the same questionnaire as 8 years previously. This time, however,
they were additionally asked whether they thought their opinions
regarding the expected benefits of D2 had changed over the inter-
vening 8 years.
Impact of ST01 results upon clinical opinion
The purpose of a clinical trial is to influence clinical opinion when
treating similar patients in future. If the results of ST01 show a
survival advantage to D2 surgery that is both statistically and
clinically significant, those surgeons with pre-study `prior' beliefs
that D2 is superior would presumably become more strongly
convinced of its efficacy whilst those who were sceptical
regarding D2 surgery would be less strongly swayed in its favour.
Statistical methods for combining prior beliefs with the observed
data from a clinical trial have been described by Fayers et al
(1997). These `Bayesian' methods enable an estimation of the
revised, or `posterior', beliefs that we would expect the surveyed
surgeons to hold after they are told the trial results.
RESULTS
Pre-study survey, 1986
Baseline survival rate for D1 patients
The average estimated value of the baseline 5-year survival for D1
patients was 21% by the three surgeons on the trial steering
committee. This agrees with the overall estimate by the total 26
participating surgeons surveyed, which was 18% (95% confidence
interval (CI) 15-22). It confirmed the initial informal pre-study
estimate of 20% for 5-year survival as a realistic assumption for
sample-size calculations.
Clinically worthwhile difference
Most clinicians indicated that a 5-10% 5-year survival advantage
to D2 would leave them uncertain whether to use D1 or D2
surgery; some chose 10-15% as equivalent, whilst a few chose
lower limit of 0 or an upper limit of 20%. The average overall
`range of equivalence' was 4.6-10.8%. The average range of
equivalence for the three surgeons of the trial steering committee
was 2-7%, suggesting that the steering committee were more
willing than other surgeons to accept a small survival benefit as
indicating that D2 was worthwhile. That is, they were enthusiasts
for the D2 treatment.
Expected difference between D1 and D2
Figure 2 shows the opinions of the 26 surgeons as to the likely
outcome of the clinical trial. The mean value for the three surgical
members of the steering committee is 13.1%. This is also above
the range of equivalence, and therefore represents a belief that D2
surgery offers a realistic and worthwhile survival benefit.
However, the larger group of surgeons was more cautious and on
average thought the survival advantage to D2 was more likely to
be 9.4%. There was considerable variation in the opinions
expressed, and the 95% CI for the mean expected survival differ-
ence was 5.6-11.0%. This represents a feeling that the results of
the trial would probably indicate a barely worthwhile advantage to
D2, since 9.4% is at the upper limit of most surgeons' range of
equivalence.
Implication for sample-size estimation
The values entered on the questionnaire by the trial steering
committee were consistent with the opinions that they had previ-
ously expressed and which had been used for the sample-size esti-
mation in ST01. The larger sample of surgeons were rather more
sceptical about expected benefits of D2, and a sample size nearly
twice as large would have been necessary if 9.4% were selected as
the target improvement in survival. More details of this technique
of assessing clinicians' opinions, together with a general review of
sample-size estimation, is available in Fayers and Machin (1995).
Sample size and clinician beliefs 215
British Journal of Cancer (2000) 82(1), 213-219
(c) 2000 Cancer Research Campaign
Second survey, 1994
Twenty-one of the original 26 surgeons, including the three
steering committee members, were traced and agreed to complete
the survey in 1994. Of the other five, one had died, one refused,
one could not be traced, and two had retired and did not feel able
to pass an opinion.
Baseline survival rate for D1 patients
The trial steering committee surgeons now thought that the base-
line survival for D1 was likely to be 32% at 5-years - an increase
of 10% over the initial beliefs 8 years earlier. However, when all
26 surgeons were included, the results showed no statistically
significant overall change of opinion (18% in 1986, 21% in 1994).
Clinically worthwhile difference
There was hardly any change in the overall range of equivalence,
which now ranged from 5.4% to 10.6%. The revised range for the
steering committee was now 6.0-10.7%, closely similar to that of
all surveyed surgeons.
Expected difference between D1 and D2
There was a small shift in the opinions of the 21 surgeons in the
later survey, from the 1986 average of 9.4% to 8.3% at the end of
the study (95% CI 7.8-11.1%). The overall results are shown in
Figure 3. However, the initial enthusiasm of the steering
committee surgeons had decreased substantially - from 13.1%
originally, to 9.5% at the end of the study. Thus the revised
opinions of the steering committee are within the bounds of the
confidence interval from the total surveyed group.
Sample-size estimation
Suppose we were designing the ST01 trial in 1995. The expected
benefit conferred by D2 would have been estimated as 9.5%.
Using a baseline of 20% 5-year survival, about 750 patients would
be necessary. However, the baseline survival rate would have been
estimated at 32%, which is substantially greater than the originally
estimated 20%. Calculations show that the power to detect an
improvement of 9.5%, from 32% 5-year survival for D1 to 41.5%
for D2, would have fallen to 54% if the study size were specified
as 400 patients. Such a low power is unacceptable. To maintain
90% power, the recruitment would have had to be extended to
1000 patients.
Results of the trial
In 1993 the ST01 gastric cancer trial completed its intended
patient accrual of 400 patients. Analysis of post-operative
mortality and morbidity confirmed that, as anticipated, there was
increased post-operative mortality associated with D2 surgery,
with 6.5% of D1 and 13% of D2 patients dying within 30 days or
without leaving the hospital (Cuschieri et al, 1996). These results
were closely similar to the findings of a Dutch randomized trial
comparing the same two operations, in which post-operative
mortality rates in patients allocated to D1 and D2 surgery were 6%
and 10%.
216 PM Fayers et al
British Journal of Cancer (2000) 82(1), 213-219 (c) 2000 Cancer Research Campaign
0.5
0.4
0.3
0.2
0.1
0.5
0.4
0.3
0.2
0.1
0.5
0.4
0.3
0.2
0.1
0.5
0.4
0.3
0.2
0.1
0.5
0.4
0.3
0.2
0.1
1* 2* 3* 4 5 6
7 8 9 10 11 12
13 14 15 16 17 18
19 20 21 22 23 24
25 26
Total
0.5
0.4
0.3
0.2
0.1
Fraction
expected 5-year difference
Prior distributions by surgeon 1986
-25
-15-5 15 25
5 -25
-15-5 15 25
5
-25
-15-5 15 25
5 -25
-15-5 15 25
5 -25
-15-5 15 25
5
-25
-15-5 15 25
5
Figure 2 Expected percentage difference in 5-year survival between D1 and D2 surgery, as predicted by 26 experienced surgeons in 1986. The overall
average results are shown to the bottom, right. Steering committee members (surgeons 1-3) are indicated*. Mean expected difference 9.4% better survival in
D2 patients, 95% confidence interval 5.6-11.0%
The 5-year survival rates have now been published (Cuschieri
et al, 1999). The overall patient survival at 5 years was 34% (95%
CI 29-39%). The two treatment arms were very similar, and D2
surgery did not appear to offer any significant benefit over D1
(hazard ratio =1.10, 95% CI 0.87-1.30). Figure 4 shows the
survival curves, and the impact of the initial post-operative
mortality seems to be largely sustained throughout the curves.
The ST01 trial 5-year survival within the D1 group was 30%,
which was closely similar to the baseline survival of 32% that was
anticipated in 1994 (second survey) by the steering committee. It
contrasts markedly with the 20% that was predicted by both the
steering committee in 1986 and by the other surgeons in 1986 and
1994.
Estimate of impact of ST01 results
The observed results can be adjusted to allow for the strong belief
that many surgeons have concerning survival advantages due to
D2 surgery. The technical details of this `Bayesian approach' are
described, with simple worked examples, in the tutorial by Fayers
et al (1997) and in Parmar et al (1994). Briefly, the survival proba-
bilities for the two treatment groups are converted into log hazard
ratios, because these are statistically more convenient for analysis.
A hazard ratio of greater than 1 indicates an advantage to D1 (as
found in the ST01 results), and this corresponds to a log hazard of
greater than zero. The prior opinions shown in Figure 3, combined
with the mean baseline estimate for the D1 survival rate (21%),
have a mean log hazard ratio of 0.232 and a standard deviation of
0.226. Applying the Bayesian methodology, we obtain Table 1 as
following:
The `uninformative prior' represents an open mind, with no
prior opinion regarding the differences between D1 and D2. This
corresponds more or less to conventional significance testing. This
analysis of the ST01 results shows that there is very little chance of
a 5% improvement, and a negligible chance of a 10% benefit. The
`enthusiastic prior' incorporates an adjustment based upon the
optimistic beliefs expressed in the 1994 survey. In principle, even
a small amount of confirmatory evidence from a clinical trial is
likely to suffice to convince an enthusiast that D2 is superior, and
substantial negative evidence would be necessary to dissuade an
enthusiast; the degree of evidence that is necessary will depend
upon the strength and range of the enthusiast's prior beliefs. The
method of calculation is illustrated in Fayers et al (1997).
Although an enthusiastic prior suffices to make it plausible that
there is a benefit to D2 that is greater than zero (probability =
0.66), it remains unlikely that there could be a 5% increase in
survival and it is highly improbable that the gain is as large as
10%.
DISCUSSION
Clinical trials are often carried out by enthusiasts who are
convinced that a new treatment is effective. It calls for consider-
able perseverance to design, seek and obtain resources, launch and
execute a multicentre randomized controlled trial. Many would
only embark upon such a course if they believed that the new treat-
ment potentially represents a major breakthrough. In view of this,
it is perhaps not surprising that the trial steering committee started
with opinions that were appreciably more optimistic than those of
their colleagues. The initial expectations of this committee
mellowed over time, and they became consistent with the more
generally expressed opinions.
The opinions of the steering committee had changed substan-
tially. Since the MRC Cancer Trials Office maintains extremely
strict confidentiality of clinical trial data until patient accrual is
completed, no hint of the results was known to anyone outside the
office until the trial was closed. Thus any modification of opinions
would be likely to be due to such influences as personal experience
with D1 and D2 surgery, prevailing national and international
opinion, publications in journals, and hearsay. Whilst we have
Sample size and clinician beliefs 217
British Journal of Cancer (2000) 82(1), 213-219
(c) 2000 Cancer Research Campaign
0.5
0.4
0.3
0.2
0.1
0
-25 -20 -15 -10 -5 0 10 15 20 25 30
Fraction
expected 5-year difference
All surveyed surgeons 1995
5
Figure 3 Expected percentage difference in 5-year survival between D1
and D2 surgery, as predicted by 21 experienced surgeons in 1995. Mean
expected difference: 8.3% better survival in D2 patients, 95% confidence
interval 7.8-11.1
1.0
0.9
0.8
0.7
0.6
0.5
0.4
0.3
0.2
0.1
0.0
0 1 2 3 4 5 6 7
Survival
Patients at risk (Events)
D1 Surgery
D2 Surgery
Events
137
144
Total
200
200
D1 Surgery
D2 Surgery
200
200
(58)
(68)
142
132
(30)
(34)
108
97
87
76
(13)
(6)
66
65
(8)
(5)
48
54
(3)
(4)
35
36
(3)
(3)
27
26
(15)
(19)
Figure 4 Survival in ST01
Table 1 Probabilities of survival benefit (increased percentage of patients
alive at 5 years following D2) being greater than the specified target
Target survival Uninformative Enthusiastic
improvement prior prior
0% 0.21 0.66
5% 0.02 0.12
10% 0.001 0.004
confidence in the results of the second survey with respect to the
steering committee members and some of the other surgeons, it is
important to bear in mind that this survey was intentionally
re-questioning the same surgeons that were surveyed 9 years
previously. Reservations must be expressed as to how realistically
the average expectations expressed in 1994 represent the
prevailing consensus opinion. Some of those included in this later
survey had retired from active work, and others were no longer
practising in this area of surgery. Also, many surgeons active in
gastric surgery in 1994 were explicitly excluded because they had
not been surveyed in 1986; some of these would have been
enthusiastic supporters for D2 surgery. Our sample was in no way
a random sample of current UK gastric surgeons.
The value of our results is in terms of their implications for trial
design. In particular, the results indicate that those involved in trial
planning will often be more optimistic about treatment effects than
their peers, and these opinions may well become modified - and
arguably more realistic - as the time progresses. Hence a trial
design committee may well aim to detect larger treatment effects
than are perhaps realistic. This can lead to a smaller sample size
than is necessary, with the attendant problems of low power to
detect treatment benefits.
There is still some confusion over the operational definition of
the alternative hypothesis in sample size calculations for clinical
trials. Spiegelhalter and Freedman (1986) discussed the confusion
between clinical demands and clinical expectations in this regard.
They pointed out that both are necessary elements in sample-size
determination but that the expectations should be the main guide
to choosing the alternative hypothesis. They quantified clinical
demands in terms of the range of equivalence. The upper limit of
this range is the minimum improvement that is considered
clinically important. Many investigators writing about sample-size
determination have advised that the alternative hypothesis repre-
sents a clinically important difference. Some have specified that it
be the smallest clinically important difference, that is, the upper
limit of equivalence. This upper limit represents the smallest
improvement of importance, and if there were sufficient power to
detect this then there would be sufficient power to detect any
important difference in the same direction. The downside to such a
suggestion is that usually the sample size based on such a policy
will be huge, and the policy would therefore tend to dissuade
researchers from starting trials. In our particular trial the non-
steering committee clinicians' opinion of the expected benefit was
actually quite close to the upper limit of equivalence, but it will not
be so in all circumstances. For trials where there is a realistic
expectation of an improvement much above the upper limit of
equivalence the policy would demand a sample size far in excess
of what is required. We therefore think it is preferable to base the
alternative hypothesis on clinicians' opinion of the likely benefit,
but taking trouble to obtain an opinion that is widely-based and not
reliant on one or two enthusiasts.
For this trial a novel method of assessing clinicians' opinions
was used, involving a detailed questionnaire answered by experi-
enced gastric surgeons. The opinions expressed in the survey were
consistent with the views of the trial planning committee, that the
trial should aim to detect a change in 5-year survival from 20%
(D1) to 33% (D2). This method proved invaluable for elucidating
clinicians' opinions, and obtaining a general feel as to the differ-
ence of interest. Clinicians voiced the opinion that it made them
think deeper about the forthcoming trial. ST01 was launched and
recruited 400 patients as originally planned.
The initial baseline estimate of 5-year survival at 20% was
incorrect. This cannot be ascribed simply to improvement in
surgical technique, since there are many other explanations that
are equally or more likely. Not all patients are entered into clinical
trials, and those recruited to ST01 may have been healthier than
initially anticipated; in particular, patients could only be random-
ized if they were fit enough to be suitable for either operation.
Medical care may have changed over the years. Cases may be
diagnosed with earlier stage disease. More-rigorous staging proce-
dures may have led to greater detection of advanced disease, with
the subsequent exclusion of poor prognosis patients. It is inter-
esting that at the second survey the trial steering committee
surgeons correctly thought that the D1 survival rate of trial patients
would be higher. This might partly be attributed to their own
experience with patients who satisfied the eligibility criteria. In
addition, although the trial results were confidential pending the
completion of accrual, summary details of pre-randomization
patient characteristics were available to the steering committee.
These included tables showing the number of patients recruited by
stage and extent of disease at presentation.
Over the 8 years that the trial was open to patient recruitment,
many surgeons modified their views about D2 surgery. By 1994,
most of those originally surveyed had come to believe that a 13%
difference was too optimistic. The consensus opinion had become
that 9-10% difference would be more realistic, and that this would
still represent a sufficiently large difference to influence future
surgical practice. Repeating the calculations for 32% survival in
D1 and 41.5% in D2 (a 9.5% improvement), over 1000 patients
would be required. This is more than double the number of
patients in the ST01 trial, and would have required another 10
years recruitment. It was decided not to extend patient recruitment.
This decision was partially based upon knowledge of a parallel
trial that was being conducted in The Netherlands, and the two
groups have agreed to carry out a joint analysis when the 10-year
survival data of the two randomized trials becomes available.
CONCLUSIONS
Decisions about sample size are one of the most important aspects
of clinical trial design. Funding bodies, ethical review committees
and many medical journals all require explicit description of pre-
study estimation of sample size and power. Many people recognize
that it is rarely of any relevance to quote a precise estimate of the
number of patients required, and it is customary to liberally round
any sample size estimates upward. Despite this, even carefully
designed trials may use sample sizes that, with hindsight, may be
seen to be unrealistically small. We suggest that those designing
trials should be circumspect about the optimism that may be
expressed by trial planning committees. One possibility is to base
sample size estimates upon a survey of clinical specialists. Maybe
the views of those on the design committee should be excluded as
being potentially biased.
For ST01, the survey of potential participants about their prior
beliefs was being tried on an experimental basis and was not used
to affect the study design. Since then, the method of surveying
clinical opinion is one that the MRC Cancer Trials Office has been
employing for an increasing number of trials, both surgical and
non-surgical. We usually survey both intending participants and
non-participants, to ensure broad coverage of opinions. It is a
method that we advocate for wider use. In our experience, clini-
cians are actively interested in participating in such exercises, and
218 PM Fayers et al
British Journal of Cancer (2000) 82(1), 213-219 (c) 2000 Cancer Research Campaign
the information collected is very helpful towards ensuring that the
trial is designed as realistically as possible. This approach, when
used for trial design, enables the opinions of those outside the
planning committee to be incorporated into the design process.
An important aspect of the approach is the recognition of a
`range of equivalence'. Although this may at first sight be thought
to be an unnecessary complication, our experience confirms that
this concept reflects the way that clinicians think about treatment
benefits. Many clinicians would find it more difficult to answer the
apparently simpler question about the level of treatment benefit is
important and worthwhile.
We recommend that both the expected changes and the range of
equivalence should be assessed in a survey when designing a clin-
ical trial. It is important to confirm that the anticipated treatment
effect would be large enough to cause a change of practice in a
reasonable proportion of clinicians, and that it would therefore be
regarded as clinically relevant. Both the expected treatment effect
and the range of equivalence should be considered for sample-size
estimation.
In addition to their implication for sample size estimation, the
elicitation of clinicians' prior beliefs can also be incorporated in a
Bayesian approach to analysis of clinical trials. This allows inter-
pretation of the results to be influenced by the optimistic - or,
possibly, sceptical - opinions of the medical community.
CONTRIBUTORS
Alfred Cuschieri was the chairman of the ST01 planning and
steering committees, from 1985 until 1997, and John Fielding and
John Craven were the two other surgical members. Peter Fayers
was study statistician from 1989 to 1997, and was responsible for
the second survey. The initial 1986 survey was the idea of
Laurence Freedman, who was also responsible for carrying it out.
Barbara Uscinska took on the difficult task of tracing the surgeons
who had participated in the initial survey, and persuading those
still alive to repeat the exercise in 1995. All of these named
persons were involved in the writing of this report.
ACKNOWLEDGEMENTS
In addition to those surgeons on the writing committee (AC, JF,
JC), the following were involved in the two surveys and we
acknowledge their help: J Banciewicz, A I M Cook*, W M
Cooke*, M J Cooper*, N J Dorricott*, G H Dunstone, D J Ellis*,
A W Hall*, R I Hall, M J Gough*, M J McMahon*, M C
Pietroni*, A Pollock*, J G Roberts, G R Sagor*, R D Stedeford*,
C Stoddard*, T V Taylor*, J G Temple, D E F Tweedle*, C M
White*, H S Winsey, R A B Wood* (*participant in 1986 and
1995 surveys;  participant in 1986 only).
REFERENCES
Cuschieri A, Fayers PM, Fielding JWL, Craven JL, Bancewicz J, Joypaul V and
Cook P (1996) Postoperative morbidity and mortality after D1 and D2
resections for gastric cancer - results of the MRC randomised controlled trial.
Lancet 347: 995-999
Cuschieri A, Weeden S, Fielding J, Banciewicz J, Craven J, Joypaul V, Sydes M,
Fayers P, for the Surgical Cooperative Group (1999) Patient survival after D1
and D2 resections for gastric cancer: long-term results of the MRC randomised
surgical trial. Br J Cancer 79: 1522-1530
Fayers PM and Machin D (1995) Sample size: how many patients are necessary?
Br J Cancer 72: 1-9
Fayers PM, Ashby D and Parmar MKB (1997) Tutorial in biostatistics: Bayesian
data monitoring in clinical trials. Stat Med 16: 1413-1430
Machin D, Campbell MJ, Fayers PM and Pinol A (1997) Sample Size Tables for
Clinical Studies, 2nd edn. Blackwell Science: Oxford
Maruyama K, Okabayashi K and Kinoshita T (1987) Progress in gastric cancer
surgery and its limits of radicality. World J Surg 11: 418-426
Parmar MKB, Spiegelhalter DJ and Freedman LS (1994) The CHART trials:
Bayesian design and monitoring in practice. Stat Med 13: 1297-1312
Spiegelhalter DJ and Freedman LS (1986) a predictive approach to selecting the size
of a clinical trial, based upon subjective clinical opinion. Stat Med 5: 1-13.
Sample size and clinician beliefs 219
British Journal of Cancer (2000) 82(1), 213-219
(c) 2000 Cancer Research Campaign

				
